# Input-Output Efficiency of Water-Energy-Food and Its Driving Forces: Spatial-Temporal Heterogeneity of Yangtze River Economic Belt, China

**DOI:** 10.3390/ijerph19031340

**Published:** 2022-01-25

**Authors:** Min Ge, Kaili Yu, Ange Ding, Gaofeng Liu

**Affiliations:** Business School, Hohai University, Changzhou 213022, China; kaili.yu@sinofaith-ip.com (K.Y.); 2063810106@hhu.edu.cn (A.D.)

**Keywords:** IOE-WEF, driving forces, spatial-temporal heterogeneity, YREB

## Abstract

The high-quality development of the Yangtze River Economic Belt (YREB) plays a crucial role in economic transformation in China. Climate change, rapid population growth, and increased urbanization have contributed towards increased pressures on the water, energy, food (WEF) nexus system of YREB. Thus, there is an imperative need to improve the efficiency of WEF in YREB. However, few studies have conducted spatial-temporal heterogeneity exploration of YREB about the input-output efficiency of WEF (IOE-WEF). Using panel data from 2008–2017, a super slack based model (SSBM), combined with the spatial autocorrelation and spatial econometric method, were proposed to calculate the IOE-WEF of YREB’s 11 provinces, the results indicated that: (1) From the perspective of time, the IOE-WEF in YREB was relatively low and displayed a fluctuating downward pattern while considering the undesirable outputs. (2) From the perspective of space, the spatial distribution of IOE-WEF in YREB was uneven. The efficiency values of the three sub-regions of YREB were “the lower reaches > the middle reaches > the upper reaches”. The IOE-WEF of YREB had a prominent positive spatial correlation and also had a spatial spillover effect. (3) The spatial aggregation effect of IOE-WEF of YREB is gradually weakening. The spatial aggregation types of IOE-WEF in YREB were “high-high” cluster areas in lower reaches and “low-low” cluster areas in upper reaches. (4) From the perspective of driving forces, environmental regulation and technological innovation promoted the improvement of IOE-WEF of YREB, while the industrial structure and mechanization level inhibited the improvement of IOE-WEF of YREB. Furthermore, the role of government support of IOE-WEF of YREB was not obvious. The improvement of IOE-WEF in adjacent regions also had a notable positive spatial spillover effect on the region.

## 1. Introduction

WEF is the material basis of the essential needs of humanity’s survival and development and is central to the sustainable development of regions and countries. In recent years, frequent natural disasters, deterioration of the ecological environment, and the sudden outbreak of the COVID-19 epidemic have brought severe challenges to human survival and development [1,2,3,4]. In response to the global crisis, China has proposed strategic objectives of “high-quality development”, “peak carbon dioxide emissions”, and “carbon neutrality” to promote the construction of a shared home for humanity, the community of common destiny [5,6,7,8]. In China, YREB passes through the eastern, central, and western plates, covering 11 provinces along the Yangtze River. Relying on the resource advantages of the Yangtze River Golden Waterway, the YREB has achieved rapid economic development and become the main force leading China’s high-quality economic development [9,10,11]. Due to its advantageous geographical location, the water and soil resources of the YREB are sufficient [12]; its total grain output accounts for more than one-third of the country [13]. In addition, the YREB is rich in mineral resources and is now one of the most concentrated areas of modern industry in China [14,15]. Over the last few years, the rapid economic growth and urbanization of the YREB induced the huge consumption of natural WEF resources and degradation of the ecosystem [16], which restricted the sustainable development of YREB [17]. Protecting YREB’s environment, rather than carrying out large-scale development, has become the key concern of the country’s river development plans. Improving the current ecological environment and achieving coordinated, green development of these economically developed provinces and municipalities of the YREB has become a crucial challenge for the green development of the YREB at the present stage [18]. The effective governance of multiple resources is related to the resource security issues under the new situation [19]. The relationship among WEF was first defined as “nexus” at the Bonn Conference in Germany in 2011, which sparked a wave of exploration on WEF nexus [20]. In fact, as early as the 1980s, relevant academic conferences and research projects were held all over the world. For example, the United Nations University (UNU) implemented a food and energy relationship research project in 1983, arguing that there is an important link between food and energy issues. The WEF nexus focuses on the interrelation among WEF, emphasizing the integration of all interrelated elements across disciplines and sectors, and considering resource management from a holistic and systematic perspective, which helps to improve the total factor resource efficiency, and also avoids the adverse impact of the single-sector resource development strategy on resources in other sectors [21,22].

With the introduction of the nexus theory, scholars have realized the importance of “nexus thinking” in promoting the effectiveness and fairness of resource management [23]. The initial research mainly focused on explaining the nexus between WEF qualitatively. For example, Hoff [16] considered the WEF nexus as a new method to promote resource utilization efficiency, reduce trade-offs between different resource management departments, and help to establish resource management synergies and improve resource governance across sectors. Compared with the integrated water resources management approach (IWRM), the nexus approach is more universal; it focuses on maximizing the efficiency of the scarce resources, improving the overall utilization efficiency of resources, generating cross-departmental benefits, and truly realizing the transformation of resource management from “integration” to “synergy” [24]. The WEF nexus can describe the interaction and interdependence between elements and also enables and supports transition and transformation across sectors and stakeholders [25]. Venghaus and Hake [26] analyzed the current policies of WEF in the EU through case analysis and explored the application of nexus thinking in reality.

With the deepening of research, the research on the WEF nexus has gradually turned to quantitative analysis. The research methods mainly include input-output optimization assessment [27,28], social network analysis [29,30], system dynamics model [31,32], coupling coordination degree model [33,34] and data envelopment analysis [35,36].

The existing research methods are comprehensive in the WEF nexus assessment. However, the inefficient use of WEF resources is still a prominent contradiction among current economic development, effective resource use, and environmental protection. Therefore, according to the development strategy of comprehensively improving resource utilization, further research on the IOE-WEF of YREB is of great significance. According to the results of efficiency analysis, the effectiveness of management can be judged, and management suggestions can be put forward. Using the DEA model, Li et al. [35] took the total consumption of WEF as direct input, the permanent population as indirect input, GDP as the expected output, and the environmental pollution index as unexpected output to measure and evaluate the IOE-WEF in 30 provinces in China. Chen et al. [36] added fixed capital as an input index and analyzed IOE-WEF and its influencing factors in 12 cities in Inner Mongolia by establishing super-efficiency SBM and Tobit model. Some scholars measured the efficiency values of each subsystem in the WEF system and tested the spatial correlation of two subsystems. It was found that there is some synergy between the efficiency values of the two subsystems [37]. Other scholars measured the IOE of agricultural resources in China based on the WEF nexus [38]. Most of the existing research focused on the national and provincial efficiency measurement and spatial-temporal differences analysis of WEF; a few studies measured the IOE of three subsystems in the WEF system and discussed the spatial correlation of the two subsystems. In addition, in the selection of input indicators, some studies took grain consumption as the direct input of the nexus system, and in the selection of output indicators, CO_2_ emissions were ignored.

The IOE-WEF is affected by many factors. In the analysis of the driving forces of IOE-WEF, the study found that the proportion of science and technology expenditures and education expenditures have a notable positive effect on the IOE-WEF in about 30 provinces in China [39]. Technological progress and infrastructure [40] and the scale of enterprises, industrial structure, openness, and mechanization level [37] have been proven to have a crucial role in facilitating the IOE-WEF of China. In the influencing factors analysis of single resource efficiency, Tang and He [41] studied the total factor energy efficiency in the YREB and found that government expenditure, economic development level, and R&D input were the main factors affecting energy efficiency. In addition, economic development level and agricultural science and technology input were also the main factors promoting the improvement of cultivated land resource efficiency in the YREB [42]. Pan et al. [43] empirically studied the impact of the environmental regulation on the IOE of water resources in the YREB and found that environmental regulation significantly promoted the improvement of the IOE of water resources. The above analysis of driving forces in the YREB showed that the current analysis paid more attention to the utilization efficiency of a single resource. Based on the existing literature, this paper selected environmental regulation, industrial structure, government support, mechanization level, and technological innovation as the influencing factors to discuss the IOE-WEF of YREB. The main contributions of the article are as follows. First, this paper used panel data of the 11 provinces in YREB as samples to evaluate the efficiency of WEF from an input-output perspective. The measurement of the IOE-WEF in YREB enriched the evaluation of the efficiency of resources exploitation in the YREB by existing research. Second, when constructing the evaluation index system of the IOE-WEF, this paper supplemented the existing index system from the perspective of nexus. Third, this paper employed a spatial auto-correlation model and spatial econometric analysis to identify the relevant driving forces by the IOE-WEF in YREB, which is more consistent with the actual situation of YREB and better than the previous analyses. The rest of this article is structured as follows. In Section 2, models are constructed. In Section 3, indicators selection and data sources are presented. In Section 4, the results are revealed. In Section 5, the conclusions are described.

## 2. Methods

### 2.1. The Super-SBM Model

We defined that there are *N* decision-making units (DMUs) in the WEF nexus system, and M inputs can produce desirable outputs of S_1_ and undesirable outputs of S_2_ [44,45], which are respectively presented by $x\in R^{M}$, $y^{g}\in R^{S1}$, $y^{b}\in R^{S2}$. $X=\left[x_{1},x_{2},\ldots ,x_{n}\right]\in R^{N\times M}$ is the input matrix, $Y^{g}=\left[y^{g1},y^{g2},\ldots ,y^{gn}\right]\in R^{S1\times N}$ and $Y^{b}=\left[y^{b1},y^{b2},\ldots ,y^{bn}\right]\in R^{S2\times N}$ is the output matrix, X>0, $Y^{g}>0$, $Y^{b}>0$, the production possibility set P can be described as follows:

(1) $$P=\left\{\left(x,y^{g},y^{b}\right)\left|x\geq X\eta ,y^{g}\leq Y\eta ,y^{b}\geq Y\eta ,\right.\eta \geq 0\right\}$$

In Equation (1), η is the intensity vector. According to the existing research theories of [46], the SBM model is constructed as follows:

(2) $$\gamma =\min\left(\frac{1-\frac{1}{M}\sum_{i=1}^{M}\frac{S_{i}^{-}}{x_{i0}}}{1+\frac{1}{S_{1}+S_{2}}\left(\sum_{r=1}^{S_{1}}\frac{S_{r}^{g}}{y_{r0}^{g}}+\sum_{r=1}^{S_{2}}\frac{S_{r}^{b}}{y_{r0}^{b}}\right)}\right)$$

$$s.t.\left\{\begin{array}{c} x_{0}=X\eta +S^{-}\\ y_{0}^{g}=Y^{g}\eta -S^{g}\\ y_{0}^{b}=Y^{b}\eta +S^{b}\\ S^{-}\geq 0,S^{g}\geq 0,S^{b}\geq 0,\eta \geq 0 \end{array}\right.$$

In Equation (2), γ represents the IOE-WEF of DMUs, 0 ≤ γ ≤ 1. Respectively, $S^{-}$, $S^{g}$ and $S^{b}$ indicate the slack in inputs, desirable and undesirable outputs. Only if γ = 1 and $S^{-}$, $S^{g}$, $S^{b}$ are all equal to 0, IOE-WEF of DMUs are on the frontier of production. However, when the IOE-WEF of DMUs are simultaneously efficient, which means the DMUs are all on the frontier of production, in that case, we will not be able to make any further comparisons. In order to further compare the efficiency values of efficient DMUs, the super--SBM (Slack-Based Model) was established [46,47,48], and the formula is as follows:

(3) $$\gamma^{*}=\min\left(\frac{\frac{1}{M}\sum_{i=1}^{M}\frac{{\bar{x}}_{i}}{x_{i0}}}{\frac{1}{S_{1}+S_{2}}\left(\sum_{r=1}^{S_{1}}\frac{{\bar{y}}_{r}^{g}}{y_{r0}^{g}}+\sum_{r=1}^{S_{2}}\frac{{\bar{y}}_{r}^{b}}{y_{r0}^{b}}\right)}\right)$$

(4) $$s.t.\left\{\begin{array}{c} \bar{x}\geq \sum_{j=1,\neq 0}^{N}\eta_{j}x_{j}\\ {\bar{y}}^{g}\leq \sum_{j=1,\neq 0}^{N}\eta_{j}y_{j}^{g}\\ {\bar{y}}^{b}\geq \sum_{j=1,\neq 0}^{N}\eta_{j}y_{j}^{b}\\ \bar{x}\geq x_{0},{\bar{y}}^{g}\leq y_{0}^{g},{\bar{y}}^{b}\geq y_{0}^{b},{\bar{y}}^{g}\geq 0,\eta \geq 0 \end{array}\right.$$

In Equation (3), $\gamma^{*}$ stands for the IOE-WEF in YREB. The range can be greater than 1. The variables in Equation (3) are the same as Equations (1) and (2).

### 2.2. Spatial Autocorrelation

#### 2.2.1. Global Moran’s I (GMI)

The global spatial autocorrelation was first proposed by Moran [49] to judge if there are obvious correlation characteristics for the research object. The formula is as follows:

(5) $$I=\frac{n\sum_{i=1}^{n}\sum_{j=1}^{n}w_{ij}\left(x_{i}-\bar{x}\right)\left(x_{j}-\bar{x}\right)}{\sum_{i=1}^{n}\sum_{j=1}^{n}w_{ij}{\left(x_{i}-\bar{x}\right)}^{2}}$$

In Equation (4), I represent the Global Moran’s I, $I\in \left[-1,\;1\right]$. I>0 means a positive spatial correlation, and I<0 indicates a negative correlation. I=0 means no spatial correlation. n is number of 11 provinces, $x_{i}$ and $x_{j}$ represent IOE-WEF of province i and province j. $\bar{x}$ is the average of IOE-WEF in YREB. $w_{ij}$ is the 0–1 weight matrix.

#### 2.2.2. Local Moran’s I

If the spatial correlation about the YREB past the GMI test, the local Moran’s I test will be calculated to recognize the spatial clusters and spatial outliers and is expressed by the Local Moran’s I [50,51]. Its calculation equation is:

(6) $$I_{i}=x_{i}\sum_{j\neq 1}^{n}w_{ij}x_{j}$$

In Equation (5), $I_{i}$ is the Local Moran’s I. if $I_{i}$ is positive, it demonstrates that the IOE-WEF of province i shows a positive correlation with that of neighboring province j, and if the value of $I_{i}$ is negative, it indicates that the IOE-WEF of province i shows a negative correlation with that of neighboring province j. n, $x_{i}$, $x_{j}$, $w_{ij}$ are same as above.

### 2.3. Spatial Econometric Model

If there are significant spatial agglomeration characteristics of the IOE-WEF in YREB under the GMI test, it needs to adopt the spatial econometric analysis to find out the crucial driving forces. In this paper, the spatial Durbin model was chosen as the general paradigm to study the driving forces of the IOE-WEF in YREB, which can be presented as follows:

(7) $$\left\{\begin{array}{c} Y=\rho Wy+\beta X+\varepsilon \\ \varepsilon =\lambda W\varepsilon +\mu \end{array}\right.$$

In Equation (6), Y represents the IOE-WEF; ρ denotes the spatial auto-regressive coefficient of the IOE-WEF; Set W as spatial weight matrix; Set X as the explanatory variables; β denotes spatial auto-regressive coefficients of the explanatory variables; ε and μ are the random error terms complying with normal distribution; Set λ as spatial error coefficient, and if λ is significantly 0, Equation (6) can be simplified to SLM; if ρ is significantly 0, Equation (6) can be simplified to SEM.

## 3. Indicators Selection and Data Processing

### 3.1. Nexus Analysis of Index

The WEF nexus system is a complex system. The system’s external environment includes the economic, social, and ecological environment. The internal system consists of three subsystems, namely water resources, energy, and food subsystems. The three subsystems interact with each other and the external environment to continuously exchange materials and energy [52,53]. From the perspective of the water resources subsystem, a certain amount of energy needs to be invested in the production and consumption of water resources, such as seawater desalination, drinking water treatment, and so on [54,55]. From the perspective of the energy subsystem, a certain amount of water resources and food need to be invested in the processing of natural gas, oil, coal, etc. Actually, the process of coal mining, processing, and refining needs plenty of water, as well as hydropower generation [56]. In addition, biomass resources mainly come from the organic matter of animals and plants [57,58]. From the perspective of the food subsystem, the main resources to be invested in food production and consumption include water, energy, chemical fertilizers, plastic film for farm use and cultivated land, etc. [38,59]. Furthermore, the production and consumption of resources are inseparable from infrastructure equipment and labor input, while the production and consumption of the three subsystems are accompanied by the production of GDP, waste gas, wastewater, and solid waste [35,37]. Therefore, according to the rationality and availability of the index, and referring to [35,38,60], the evaluation indexes of IOE-WEF in YREB were selected, as shown in Table 1.

#### 3.1.1. Input Indicators

Water is an important resource for human survival and economic and social development. Total water consumption was chosen to represent the input indicator of the water resources subsystem in the WEF nexus system. Total energy consumption was chosen as the input indicator of the energy subsystem. Food production requires the input of agricultural chemical fertilizers, agricultural plastic films, and cultivated land; therefore, they were considered the input indicators of the food subsystem. Employed people provide labor for regional economic development, so the number of people employed in each province was selected as the labor input. The production and consumption of resources in the WEF nexus system cannot be separated from the investment in fixed assets; therefore, the fixed assets investment of the whole society was taken as the capital input of the WEF nexus system of the YREB.

#### 3.1.2. Output Indicators

Regional GDP and environmental pollutants are the desirable and undesirable output in the WEF nexus system, respectively. On the one hand, regional GDP can be used to characterize the economic benefits of the WEF nexus system. On the other hand, waste gas, wastewater, and solid wastes will inevitably be generated in the process of material exchange and energy transfer of the WEF nexus system. Therefore, CO_2_, SO_2_, oxynitride, smoke (or dust), sewage, general industrial solid wastes are taken as the environmental costs of the WEF nexus system.

### 3.2. Data Processing

Individual indicators are dealt with as follows:

(1) According to the GDP deflator released by the National Bureau of Statistics, prices in 2008 are used as the base period to convert the total social fixed asset investment and regional GDP into real prices to increase the comparability of data.
(2) The six indicators, CO_2_ emissions, SO_2_ emissions, oxynitride, smoke (or dust) emissions, sewage emissions, and general industrial solid waste production, are selected as indicators to measure the environmental pollution situation. First, normalize the different types of indicators and then use the arithmetic mean method to construct the environmental pollution index. The standardized treatment method is as follows:
  (8) $$X_{ij}^{\prime }=\frac{X_{ij}-\min(X_{i})}{\max(X_{i})-\min(X_{i})}$$
  (9) $$G=\frac{1}{n}\sum X_{ij}^{\prime }$$

In Equation (7), $X_{ij}$ represents the value of indicator i in year j; max ($X_{i}$) denotes the maximum of indicator *i* over the study period, and min ($X_{i}$) denotes the minimum value. Firstly, the indicator is normalized by using Equation (7), and then the different indicators are normalized by using the arithmetic mean value. As shown in Equation (8), G is the environmental pollution indicator, and $X_{ij}^{\prime }$ is the normalized indicator value.

(1) According to reference [61,62], the method in IPCC (2006) was adopted to estimate the data of CO_2_ emissions of 11 provinces of YREB from 2008 to 2017.

## 4. Results Analysis

### 4.1. Spatial-Temporal Characteristics of the IOE-WEF in YREB

MaxDEA 8 software was used to calculate and analyze the spatial-temporal evolution characteristics of the IOE-WEF of 11 provinces in the YREB from 2008 to 2017.

#### 4.1.1. Time Evolution of the IOE-WEF in YREB

**Figure 1:**
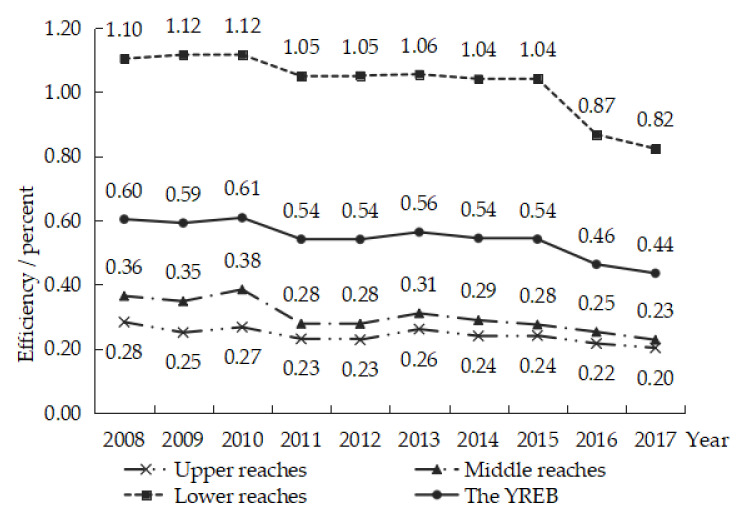
Comparison of the IOE-WEF in different regions of the YREB.

YREB is composed of upper, middle, and lower reaches. The upper reaches include Guizhou province, Sichuan province, Yunnan province, and Chongqing city. The middle reaches are comprised of Hunan and Jiangxi. The lower reaches include Anhui, Jiangsu, Shanghai, and Zhejiang. The time-varying trend of the IOE-WEF in YREB is described in Figure 1.

As shown in Figure 1, there are two temporal characteristics of the IOE-WEF in YREB.

(1) The IOE-WEF in YREB was relatively low. The average value of IOE-WEF in YREB fluctuated in the range of [0.4–0.6], which did not reach DEA efficiency. It indicated that the comprehensive resource management and industrial-scale needed to be improved in the YREB. Regionally, the efficiency values of the upper reaches fluctuated in the range of [0.2–0.3], and the efficiency values of the middle reaches fluctuated in the range of [0.2–0.4]; neither of them was DEA effective, while the efficiency value of the lower reaches was higher than that of the upper reaches and middle reaches. The efficiency value of the lower reaches reached DEA efficiency from 2008 to 2015 and had an obvious downward trend from 2016 to 2017.
(2) The IOE-WEF in YREB displayed a fluctuating downward pattern when considering the undesirable outputs. In Figure 1, the IOE-WEF all had an obvious downward trend from 2008 to 2017. It shows that during this period, the development of the YREB presented the characteristics of extensive economy and resource utilization mode. It should be noted that the efficiency values of the lower reaches showed a significant downward trend during 2016–2017. The main cause of this situation is that the IOE-WEF of Zhejiang province decreased significantly from 2016 to 2017, which reduced the average efficiency level of the lower reaches.

#### 4.1.2. Spatial Characteristics of the IOE-WEF in YREB

**Figure 2:**
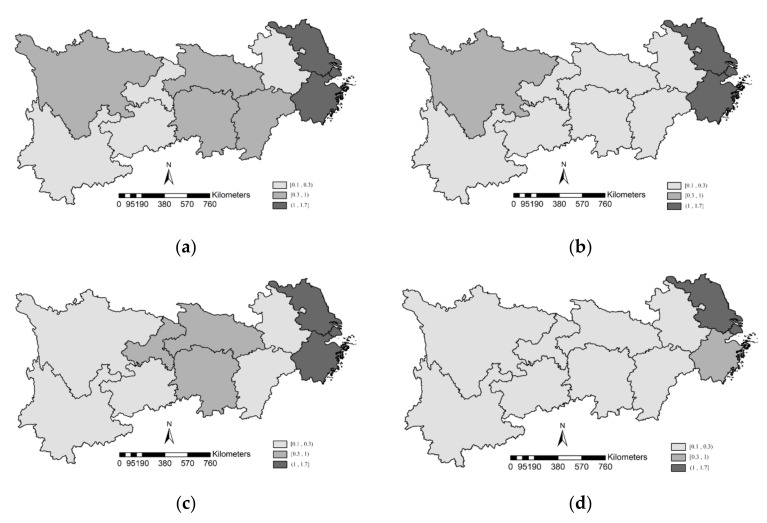
The spatial characteristics of the IOE-WEF of YREB in 2008, 2011, 2014, and 2017. (**a**) the spatial distribution of the IOE-WEF of YREB in 2008; (**b**) the spatial distribution of the IOE-WEF of YREB in 2011; (**c**) the spatial distribution of the IOE-WEF of YREB in 2014; (**d**) the spatial distribution of the IOE-WEF of YREB in 2017.

In order to further explore the spatial distribution of IOE-WEF in the YREB, ArcGIS10.0 software was used to present the spatial distribution of the IOE-WEF of YREB in 2008, 2011, 2014, and 2017, as shown in Figure 2. Referring to the existing literature, the IOE-WEF values can be classified into the high-efficiency zone [1.0, 1.7), medium-efficiency zone [0.3–1.0), and low-efficiency zone [0.1–0.3).

(1) In Figure 2, the spatial characteristics of the IOE-WEF in YREB was uneven, and the efficiency values of the three sub-regions of YREB were along with “the lower reaches > the middle reaches > the upper reaches”. Actually, in Figure 2, the IOE-WEF in the upper and middle reaches of the YREB were generally low, and the efficiency values in both reaches dropped to the low-efficiency zone over time. Sichuan, Hubei, Hunan, and Jiangxi provinces declined from the medium efficiency zone to the low-efficiency zone over time.
(2) The upper reaches of the YREB are rich in natural resources with a large water flow gap, and most of the water-energy resources are concentrated in the upper reaches. However, the extensive economic growth mode based on agriculture and animal husbandry causes serious soil erosion and damage to the ecological system in the upper reaches. In addition, silt and pollutants from the upper reaches of the river flow through the middle and lower reaches, affecting the ecological environment of the middle and lower reaches of the YREB. Therefore, relying on the consumption of large amounts of resources to promote economic development is an unsustainable way of development, and the upper reaches of the YREB still need to be improved in terms of industrial layout and rational allocation of resources.
(3) The IOE-WEF of Jiangsu province and Shanghai city have been high, while Zhejiang province dropped to the medium efficiency zone in 2017, as shown in Figure 2d. Actually, in Zhejiang, from 2013 to 2017, the growth rate of fixed-asset investment was higher than that of the regional GDP province. Especially in 2016, fixed asset investment in Zhejiang province increased by 9.6%, while regional GDP only increased by 9.0% year on year, indicating that the economic growth of Zhejiang province was more dependent on fixed capital investment. Therefore, the IOE-WEF of Zhejiang province showed a downward trend, with a significant decline in 2016 and 2017.
(4) Anhui province has been in the low-efficiency zone. Although Anhui province is in the lower reaches of YREB and adjacent to the developed coastal areas, its IOE-WEF has not benefited from the radiation effect of Jiangsu province and Zhejiang province, and the IOE-WEF has been in an inefficient state.

### 4.2. Spatial Correlation Analysis of the IOE-WEF in YREB

The IOE-WEF in YREB is spatially blocky, so the efficiency changes among provinces may be spatially correlated. Using GeoDa software, the GMI of the IOE-WEF in YREB from 2008 to 2017 was calculated to explore whether it has an obvious spatial correlation. The results are presented in Table 2.

The IOE-WEF of YREB had a notable positive spatial correlation, and its spatial aggregation effect is gradually weakening. In Table 2, the GMI of the IOE-WEF in YREB during 2008–2017 all passed the significance test. The Moran’s I measurement results were all positive, indicating a strong positive autocorrelation in the YREB. In addition, the GMI showed a “fluctuating” decreasing trend during 2008–2017. In 2010, the spatial aggregation degree of IOE-WEF in the YREB reached the peak, with the GMI of 0.620, and then showed a decreasing trend year by year. The reason for this phenomenon might be that since the “12th Five-Year” Plan, the growth rate of fixed capital investment in each province of YREB has been higher than that of economic growth, and the investment efficiency was low. As a result, the IOE-WEF in the YREB has been reduced, which leads to the gradual weakening of the spatial aggregation effect.

**Figure 3:**
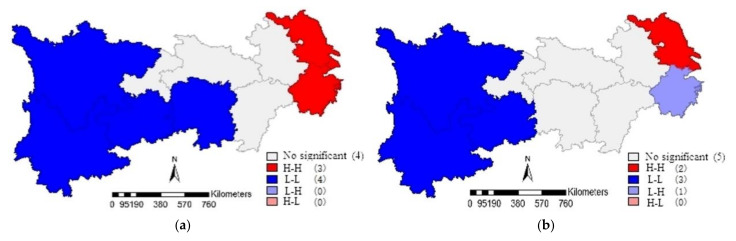
LISA aggregation diagram of IOE-WEF in 2008 and 2017. (**a**) LISA aggregation diagram of IOE-WEF in 2008; (**b**) LISA aggregation diagram of IOE-WEF in 2017.

According to the above global autocorrelation test, the IOE-WEF has a significant spatial correlation in the YREB. On this basis, the local autocorrelation test was further carried out, and the spatial aggregation types of each province could be obtained through Local Moran’s I analysis, namely “low-low”, “high-high”, “low-high”, “high-low”. LISA aggregation diagram can directly reflect the spatial aggregation areas that have passed the significance test. Taking 2008 and 2017 as examples, LISA aggregation diagram of IOE-WEF in the YREB is drawn with the help of GeoDa software, as shown in Figure 3.

(1) In Figure 3, the spatial aggregation types of the IOE-WEF in the YREB were mainly “high-high” and “low-low”. The “high-high” clustering type occurred in the lower reaches of the YREB, while the “low-low” clustering type occurred in the upper reaches of the YREB.
(2) Actually, in 2008, seven provinces passed the significance test, among which Jiangsu, Shanghai, and Zhejiang were “high-high” clusters, while Sichuan, Yunnan, Guizhou, and Hunan were “low-low” clusters.
(3) In 2017, six provinces passed the significance test, with Jiangsu and Shanghai as “high-high” clusters, Zhejiang as “low-high” clusters, and Sichuan, Yunnan, and Guizhou as “low-low” clusters.
(4) Combined with LISA aggregation maps of other years, it could be seen that Jiangsu, Shanghai, and Zhejiang were basically stable in “high-high” aggregation areas, and the IOE-WEF between provinces had a positive spatial spillover effect.
(5) Under the promotion of integration policy of the Yangtze River Delta, the Yangtze River Delta region has continued to cooperate and exchange, forming a good situation of mutual promotion and coordinated development. However, Anhui province, as a member of the Yangtze River Delta region, has not shown a significant spatial aggregation effect, so it still needs to strengthen the exchange and cooperation with other provinces further.
(6) Sichuan and Yunnan were basically stable in the “low-low” aggregation area, the two provinces with large tourism resources are adjacent to each other and have similar types and richness of resources. For the past few years, with the development of the economy, the demand for resources in the two provinces has greatly increased; coupled with the weak consciousness of government and residents to save, their ecological environment has been damaged to varying degrees.

### 4.3. Analysis of Driving Forces of IOE-WEF

#### 4.3.1. Variable Selection and Indicator Description

The driving forces affecting the IOE-WEF in the provinces of YREB are complex and diverse. Based on the existing studies, according to the availability of data, driving forces were chosen from the four aspects of environment, economy, society, and technology, which are presented in Table 3.

Specific indicators of Table 3 are described as follows:

(1) Environmental regulation (ER). Environmental regulation was represented by the proportion of industrial pollution control investment in GDP. From one side, due to the pressure of environmental regulation, the government and companies would invest more resources in environmental protection and waste treatment, which would help improve the technical level of resource utilization and pollutant treatment. On the other side, environmental regulation might increase the production cost of government and enterprises, which was not conducive to the improvement of IOE-WEF.
(2) Industrial structure (IS). The industrial structure was characterized by the proportion of tertiary industry. Industrial structure has a crucial influence on the IOE-WEF.
(3) Government support (GS). Government support was characterized by the proportion of fiscal expenditure on science and education. Government expenditure on science and education helps to promote social progress and improve the overall quality of the population, which in turn improves the IOE-WEF.
(4) Mechanization level (ML). The level of mechanization directly affects the efficiency of agricultural production; the higher the level of agricultural mechanization, the more efficient the use of resources.
(5) Technological innovation (TI). Technological innovation was represented by the number of patents granted, the progress of scientific and technological level can contribute to the improvement of enterprise production efficiency and pollution control level.

#### 4.3.2. Selection of Spatial Econometric Model

The above exploration showed that IOE-WEF of YREB had obvious spatial aggregation. Therefore, the spatial effect needs to be verified. Before conducting the spatial econometric regression, the VIF test was conducted on the explanatory variables. The spatial econometric results showed the largest explanatory variable, VIF, was 2.62, which could exclude the multicollinearity among the explanatory variables. Then, in order to screen the appropriate spatial econometric models, the LM and robust LM tests were conducted, shown in Table 4.

In Table 4, LM-lag and LM-error both passed the hypothesis test, Robust LM-lag passed the hypothesis test, and Robust LM-error did not pass the hypothesis test. Therefore, the spatial lag model (SLM) could better reflect the influence of driving forces on the IOE-WEF in YREB. In addition, based on the Hausman test results, the hypothesis of random effect was rejected at a 1% significance level. Therefore, the fixed-effects model of SLM was used. The results are revealed in Table 5.

As shown in Table 5, environmental regulation and technological innovation promoted the improvement of IOE-WEF of YREB, while the industrial structure and mechanization level inhibited the improvement of IOE-WEF of YREB. Furthermore, the role of government support of IOE-WEF in the YREB was not yet obvious. The improvement of IOE-WEF in adjacent regions also had a significant positive impact on the region.

(1) The regression coefficient of environmental regulation was significantly positive, showing that environmental regulation had a significant contribution to the IOE-WEF of YREB. The increase of investment in industrial pollution treatment implied that more capital, technology, and talents would flow into industrial pollutant treatment, which could not only improve the utilization efficiency of WEF through recycling, but also contribute to ecological, environmental protection.
(2) The regression coefficient of technological innovation was prominently positive, revealing that technological progress had a significant contribution to the IOE-WEF. With the increase in the number of patents granted, more and more patents were transformed into advanced technologies and productivity, which greatly improved the utilization efficiency of WEF.
(3) The regression coefficient of the industrial structure was notably negative, indicating that industrial structure had a negative impact on IOE-WEF. At present, the tertiary industry in the YREB is still dominated by high-consumption industries such as wholesale and retail, restaurants, and accommodation and has not yet fully realized the transformation and upgrading to high-tech service industries [63]. Optimizing the industrial structure and improving the IOE-WEF still need to be improved.
(4) The regression coefficient of mechanization level was markedly negative, illustrating that mechanization level had a significant negative effect on the IOE-WEF. The reason might be that agricultural production is still dominated by small-scale family operations in China, with less use of large and medium-sized agricultural machinery. In addition, with the outflow of a large amount of agricultural population and the reduction of arable land, fewer and fewer people are engaged in agricultural production, and a large number of agricultural machinery are abandoned.
(5) The regression coefficient of government support was not significant. It demonstrated that the effect of government support on IOE-WEF was not significant, which was different from the results of [39]. The reason might be that the difference in government support between the region and the adjacent region made the spillover effect smaller. In addition, it took a certain amount of time for the cultivation of talents and the overall improvement of the quality of residents. Therefore, there might be a time lag in the improvement of the IOE-WEF by the input of science and education. Scholars believed that excessive support from the government would disrupt the market order and reduce the efficiency of resource allocation and utilization. In addition, excessive support from the local government would attract the surrounding production factors to the local area and hinder the development of surrounding areas [64,65].
(6) The regression coefficient ρ of spatial lag term of the explained variable was 0.887, and was significantly non-zero, which verified the spatial correlation of IOE-WEF in adjacent regions of YREB, that was, for every 1% increase of IOE-WEF in the adjacent areas, the IOE-WEF in the region would increase by 0.887%.

## 5. Conclusions

From the perspective of improving resource utilization efficiency, this paper regarded WEF as a whole, conducted multidimensional analysis on the spatial-temporal characteristics of IOE-WEF in YREB, and analyzed the driving forces. The results indicate that:

(1) From the aspects of time series, the IOE-WEF in YREB was relatively low. The average value of the IOE-WEF in YREB fluctuated in the range of [0.4–0.6], which did not reach DEA efficiency. Regionally, the efficiency values of the upper and lower reaches in the YREB were low, and neither of them was DEA effective. What is more, the IOE-WEF in YREB displayed a fluctuating downward pattern when considering the undesirable outputs. Especially in 2016 and 2017, the efficiency values of the lower reaches showed a significant downward trend.
(2) The spatial distribution of IOE-WEF in YREB was uneven, and the efficiency values of the three sub-regions of YREB were “the lower reaches> the middle reaches > the upper reaches”. Sichuan, Hubei, Hunan, and Jiangxi provinces declined from the medium efficiency zone to the low-efficiency zone over time. The IOE-WEF of Jiangsu province and Shanghai city have been at a high level, while Zhejiang province dropped to the medium efficiency zone in 2017, and Anhui province has been in the low-efficiency zone.
(3) The IOE-WEF of YREB had a prominently positive spatial correlation, and its spatial aggregation effect is gradually weakening. The Moran’s I measurement results were all positive, and all passed the 1% significance test from 2008 to 2017. In 2010, the Global Moran’s I of IOE-WEF in the YREB reached its maximum, illustrating that the spatial aggregation effect of IOE-WEF in the YREB was the strongest in 2010. Then the GMI showed a decreasing trend. By the local auto-correlation test, the spatial aggregation types of the IOE-WEF in the YREB were mainly “high-high” and “low-low”, and the “high-high” clustering type occurred in the lower reaches of the YREB, while the “low-low” clustering type occurred in the upper reaches of the YREB.
(4) The improvement of environmental regulation and technological innovation was the key to promoting the IOE-WEF of YREB. The improvement of industrial structure, government support, and mechanization level had not yet become an effective help to promote the improvement of IOE-WEF of YREB. In addition, the role of government support of IOE-WEF in the YREB was not yet obvious. Finally, the IOE-WEF of YREB has an obvious spatial spillover effect, and the optimization of IOE-WEF in adjacent regions also has a positive effect on the local area.

## Figures and Tables

**Table 1:** Indicators of the IOE-WEF in YREB.

Indicator Type	Indicators	Variable	Indicator Unit
Input indicators	Resource inputs	Water consumption	10^8^ m^3^
		Energy consumption	10^4^ tce
		Consumption of chemical fertilizer	10^4^ Ton
		Consumption of agricultural plastic film	10^4^ Ton
		Total sown area of crops	10^4^ hectare
	Labor input	Employment population	10^4^ person
	Capital inputs	Fixed assets investment	10^8^ yuan
Desirable outputs	Economic benefits	Regional GDP	10^8^ yuan
Undesirable outputs	Environmental costs	Environmental pollution index	%

Note: The data are all from China Statistical Yearbook (2009–2018), China Energy Statistical Yearbook (2009–2018), China Statistical Yearbook on Environment (2009–2018).

**Table 2:** GMI measurement results of the IOE-WEF in YREB during 2008–2017.

Year	Moran’s I	Z Value	Prob.
2008	0.611	3.961	0.003
2009	0.608	3.953	0.002
2010	0.620	4.029	0.002
2011	0.610	4.011	0.002
2012	0.606	4.007	0.002
2013	0.593	3.942	0.002
2014	0.585	3.945	0.002
2015	0.583	3.920	0.002
2016	0.417	3.561	0.002
2017	0.366	3.382	0.002

**Table 3:** Driving forces of the IOE-WEF in YREB.

Driving Forces Classification	Driving Forces	Variable	Variable Symbol
Environment	Environmental regulation	Environmental pollution control investment /GDP	ER
Socio-economy	Industrial structure	Value-added of tertiary industry/GDP	IS
	Government support	Science and education expenditures/fiscal expenditures	GS
	Mechanization level	Total power of agricultural machinery	ML
Technology	Technology innovation	Number of patents granted	TI

Note: The data is from China Statistical Yearbook (2009–2018), China Energy Statistical Yearbook (2009–2018), China Statistical Yearbook on Environment (2009–2018).

**Table 4:** Spatial econometric model fitness test.

Test	LM-Lag	Robust LM-Lag	LM-Error	Robust LM-Error
LM	30.559 ***	4.934 **	26.724 ***	1.100
*p*-value	0.000	0.026	0.000	0.294

Note: ***, **, and * denote the significance levels about 1%, 5%, 10%, respectively.

**Table 5:** Estimation results of SLM model of IOE in YREB.

Variable	Coef.	Std. Err.	t	*p* > |t|
In(*ER*)	0.115 ***	0.028	4.168	0.000
In(*IS*)	−0.234 *	0.129	−1.816	0.069
In(*GS*)	−0.159	0.116	−1.378	0.168
In(*ML*)	−0.0776 ***	0.022	−3.540	0.000
In(*TI*)	0.067 ***	0.015	4.384	0.000
ρ	0.887 ***			
*δ* ^2^	0.019			
Log-*likelihood*	37.578			
Hausman	140.83 ***			

Note: ***, **, and * denote the significance levels about 1%, 5%, 10%, respectively.

## Data Availability

The data presented in this study are openly available from the National Bureau of Statistics.
